# Identification, Characterization, and Formulation of a Novel Carbapenemase Intended to Prevent Antibiotic-Mediated Gut Dysbiosis

**DOI:** 10.3390/microorganisms7010022

**Published:** 2019-01-16

**Authors:** Sheila Connelly, Todd Parsley, Hui Ge, Michael Kaleko

**Affiliations:** 1 Synthetic Biologics, Inc., Rockville, MD 20850, USA; mkaleko@syntheticbiologics.com; 2 SynPhaGen, LLC, Rockville, MD 20850, USA; tparsley@noblelifesci.com; 3 AscentGene, Inc., Gaithersburg, MD 20878, USA; hge@ascentgene.com

**Keywords:** carbapenemase, beta-lactamase, antibiotics, dysbiosis, microbiome, antibiotic resistance

## Abstract

Antibiotics can damage the gut microbiome leading to opportunistic infections and the emergence of antibiotic resistance. Microbiome protection via antibiotic inactivation in the gastrointestinal (GI) tract represents a strategy to limit antibiotic exposure of the colonic microbiota. Proof of concept for this approach was achieved with an orally-administered beta-lactamase enzyme, SYN-004 (ribaxamase), that was demonstrated to degrade ceftriaxone excreted into the GI tract and protect the gut microbiome from antibiotic-mediated dysbiosis. Ribaxamase efficiently degrades penicillin and cephalosporin beta-lactam antibiotics, but is not active against carbapenems. To expand this microbiome protection strategy to include all classes of beta-lactams, three distinct carbapenemases were evaluated for manufacturability, antibiotic degradation spectrum, and stability in human intestinal fluid. *E. coli* production strains were generated for P2A, a novel metallo-enzyme isolated from *B. cereus*, New Delhi metallo-beta-lactamase (NDM), and *Klebsiella pneumoniae* carbapenemase (KPC). While all three enzymes effectively inactivated a broad range of antibiotics, including penicillins, most cephalosporins, and carbapenems in vitro, only P2A retained biological activity when incubated with human chyme. As functional stability in the intestinal tract is a key requirement for an orally-delivered enzyme, P2A was chosen as a potential clinical candidate. An enteric formulation of P2A was developed, called SYN-006, that was inert under high acid conditions, with enzyme dissolution occurring at pH > 5.5. SYN-006 has the potential to expand microbiome protection via antibiotic inactivation to include all classes of beta-lactam antibiotics.

## 1. Introduction

The gut microbiome, defined as the collective genomes of microbiota inhabiting the gastrointestinal (GI) tract, functions symbiotically with its host for maintenance of health. Antibiotics can disrupt this complex bionetwork, causing dysbiosis, an alteration of microbiome composition and function, and lead to overgrowth of adventitious pathogens such as *Clostridium difficile*. Antibiotic-mediated changes to the gut microbiome may persist indefinitely [1,2,3] and have been associated with adverse afflictions including autism, cancer, diabetes, and obesity [4]. An immediate consequence of antibiotic exposure is that selective pressure accelerates the evolution of antibiotic resistance with the gut microbiome supplying a vast reservoir of resistance genes. Therefore, protection of the gut microbiome from antibiotic collateral damage should mitigate both the short- and long-term consequences of dysbiosis.

Antibiotic inactivation in the GI tract represents a promising strategy to protect and maintain the gut microbiota. One approach uses SYN-004 (ribaxamase), an engineered class A serine beta-lactamase enzyme formulated for oral administration, to degrade certain intravenous (IV) beta-lactam antibiotics in the GI tract to preserve the gut microbiome [5]. Verification of ribaxamase utility was obtained in animal and human studies by demonstrating that ribaxamase degraded ceftriaxone excreted into the upper GI tract, protected the gut microbiome from antibiotic damage, and limited emergence of antibiotic resistance [5,6,7,8,9]. Proof-of-concept for ribaxamase efficacy was established in a Phase 2b clinical study that met its primary endpoint of significantly reducing *C. difficile* infection in patients treated with IV ceftriaxone [7,10].

Ribaxamase efficiently degrades beta-lactam antibiotics including penicillins and most cephalosporins [5], but does not inactivate carbapenems. Carbapenems are considered a last resort therapeutic, used sparingly in humans and banned for use in food animals [11]. Such measures are intended to prolong antibiotic utility by reducing emergence of carbapenem resistance. Despite these interventions, the use of carbapenems [12] and the number of resistant infections [13] are escalating globally, so much so that the CDC have declared carbapenem-resistant Enterobacteriaceae (CRE) an “urgent threat” [14]. In addition, carbapenem use poses a strong risk for development of *C. difficile* infection (CDI) [15,16], a common health-care associated infection in the United States [17] responsible for 29,000 deaths in 2011 [18]. Therefore, protection of the gut microbiome from all types of beta-lactam antibiotics, including carbapenems, is expected to mitigate antibiotic-mediated collateral damage, decrease infection by opportunistic pathogens, and reduce the risk of antibiotic resistance.

To expand microbiome protection to all classes of beta-lactam antibiotics, three carbapenemases, chosen based on reported broad spectrum antibiotic degradation profiles, were characterized. The carbapenemases included P2A, a novel metallo-enzyme isolated from *B. cereus* (previously named targeted recombinant beta-lactamase 2 [19]), New Delhi metallo-beta-lactamase (NDM) [20], and *Klebsiella pneumoniae* carbapenemase (KPC) [21]. The enzymes were screened for manufacturability in *E. coli* production cell lines, antibiotic degradation spectrum, and retention of biological activity in human intestinal fluid. P2A was chosen as the candidate with the most potential for in vivo efficacy.

## 2. Materials and Methods

### 2.1. Plasmid Construction

Plasmids for protein production in *E. coli* were generated for P2A (targeted recombinant beta-lactamase 2) [19], NDM-1 [20] and KPC-1 [21]. A total of 39 plasmids and 104 bacterial strains were produced. For P2A, 3 gene variants, 9 plasmids and 25 bacterial strains, for NMD-1, 8 gene variants, 17 plasmids, and 44 bacterial strains, and for KPC-1, 7 gene variants, 13 plasmids and 35 bacterial strains were generated and tested. The gene expression constructs differed by plasmid backbone, expression cassette promoter, leader sequence, the N-termini of the carbapenemase coding region, and the bacterial host strain [22]. Plasmid integrity was verified by DNA sequencing.

### 2.2. E. coli Strain Enzyme Expression Screening

The *E. coli* production strains were assessed for bacterial growth and carbapenemase expression in two rounds of shake flask fermentation (3 mL and 25 mL) screening assays. Bacterial colonies were grown overnight and 1 mL of each was lysed with BugBuster protein extraction reagent (EMD Millipore), and soluble and insoluble fractions analyzed using sodium dodecyl sulfate polyacrylamide gel electrophoresis (SDS-PAGE). Because the metallo-beta-lactamases, P2A and NDM, require zinc ions for activity [23], 0.1 mM ZnSO_4_ was added to the bacterial growth media. Cell lysates from bacterial strains showing the highest protein expression levels by SDS-PAGE were evaluated for biological activity using CENTA as a chromogenic substrate for beta-lactamase activity [24,25]. Briefly, the assay was performed in a 50 mM NaH_2_PO_4_ buffer, pH 7.0 with supplementation of 0.1 mM ZnSO_4_, with CENTA (Calbiochem) at 50 µg/mL. Assays used purified SYN-004 (ribaxamase) protein [5] for the standard curve at protein concentrations of 0, 3, 6, 8, 10, 15, 20 and 40 ng/mL. The plates were read at 405 nm after a 15 incubation using a microtiter plate reader. Specific activities were calculated using the SoftMax Pro software to apply a linear fit.

### 2.3. Beta-Lactamase Production and Purification

The *E. coli* strains that displayed the highest biological activities for each enzyme were selected for scale-up in 5-liter fermenters. Biological activity was determined using the CENTA assay with purified ribaxamase protein as the positive control. Notably, the highest expressing cell lines contained expression cassettes with the *phoA* promoter [26] driving expression of the carbapenemase genes. The *phoA* promoter is repressed by high phosphate levels in media, but is autoinduced when phosphate is depleted [26]. Therefore, low phosphate bacterial growth media was used, with a 50% glucose, 2 mM ZnSO_4_ feed, salt supplement of 1 M MgSO_4_ when the OD_600_ reached ~40, and 0.1 mM ZnSO_4_ supplementation of P2A and NDM fermenters.

For enzyme purification, frozen cell pellets retained from the fermenter studies were lysed at 3 × 7000 psi in a Panda table top cell homogenizer. Insoluble material was removed by centrifugation, supernatants collected and pH adjusted to 5.5 with 1 M 2-ethanesulfonic acid (MES). The supernatants were centrifuged again, to remove precipitated debris and filtered through a 0.45 µm filter. The filtered supernatants were subjected to cation-exchange chromatography using an SP-sepharose column. NDM was subjected to an additional purification step using a hydrophobic interaction chromatography with phenyl sepharose. Fractions containing the peak levels of protein were concentrated and dialyzed against a 20 mM HEPES, pH 7.5, 150 mM NaCl buffer. The P2A and NDM samples were further supplemented with 0.1 mM ZnSO_4_ in all steps of the purification process. Purified enzymes were assessed by SDS-PAGE and biological activity verified by CENTA analysis using purified ribaxamase as the positive control.

### 2.4. Antibiotic Degradation Assay

The antibiotic inactivation activities of purified P2A, NDM, and KPC, were evaluated using a bacterial growth microtiter plate assay [5]. The assay was performed by mixing 10, 100 or 1000 µg/mL of the indicated antibiotic with purified P2A, NDM, KPC, or ribaxamase [5] at concentrations of 10, 100, or 1000 ng/mL in a 96 well microtiter plate. *E. coli* (ATCC 25922) from an overnight culture was added the wells immediately after the addition of the beta-lactamase enzymes, and the plates were incubated overnight. The assay included negative control wells of media alone and positive control wells for bacterial growth. Bacterial growth was quantified by measuring the absorbance at 625 nm (OD_625_) in a Spectramax 384 Plus plate reader. The analysis was performed twice for each antibiotic. Beta-lactamase activity was determined as positive or negative based on the appearance of bacterial growth in the individual wells. An OD_625_ of 0.8 or greater indicated maximal bacterial growth and, therefore, equated with complete antibiotic degradation by the beta-lactamase. An OD_625_ of less than 0.8 indicated lower bacterial growth therefore incomplete antibiotic degradation, hence lower beta-lactamase activity.

### 2.5. Beta-Lactamase Antibiotic Degradation Kinetics

The antibiotic degradation kinetics of ribaxamase and P2A were determined by measuring substrate hydrolysis under initial rate conditions with Hanes linearization [27] of the Michaelis–Menten equation. The reactions were performed in 20 mM phosphate buffer (pH 7.0) at 30 °C. Briefly, dilutions of each antibiotic were prepared in sodium phosphate buffer and distributed to individual wells of a 96-well plate. The plate was pre-incubated at 30 °C for 5 min, after which 1.0 nM enzyme was added to the plate. The absorbance at 235 nm (ampicillin and piperacillin), 257 nm (ceftriaxone), 264 nm (cefotaxime, cefazolin, cefoperazone, cefuroxime), or 300 nm (imipenem and meropenem) of the individual wells was determined every 5 to 8 seconds over a 10- to 20-minute period of incubation. The initial velocity was calculated as the change in absorbance per min (mU_abs_/min) of the reaction as determined using SoftMax Pro 5.4 software from the slope of the curve within the linear range of the reaction. Following the kinetic analysis, an endpoint reading was taken to determine the pathlength (cm) of the specimen in each individual well. The velocity (mU_abs_/min) values were normalized to a 1 cm pathlength by dividing the values by the measured pathlength. The normalized velocity values (mU_abs_/min-cm) were then converted to velocity (nmole/sec-cm) using an experimentally determined extinction coefficient specific for each individual antibiotic at the given wavelength. The data were imported into Prism GraphPad 5 for determination of Michaelis-Menten kinetics by non-linear regression analysis.

### 2.6. Human Intestinal Chyme Enzymatic Activity Stability Analyses

Human intestinal chyme, collected from five donors with ileostomies who signed an informed consent, was obtained from the Oklahoma Foundation for Digestive Research (OFDR) under a University of Oklahoma Institutional Review Board (IRB) approved protocol. Chyme samples were characterized by pH, liquid content, and protease activity (Table S1). Intrinsic proteolytic activity of the chyme specimens was determined using a commercial Protease Activity Fluorometric Assay Kit (Biovision, Milpitas, CA, USA) following the manufacturer’s recommended procedures. Chyme specimens were diluted 1:20 in protease assay buffer prior to performing the protease assay. The mixed chyme sample contained equal volumes of each of the five chyme specimens.

P2A, NDM, and KPC proteins (final concentration of 80 ng/mL) were added to mixed chyme or CENTA assay buffer and samples were then collected over a 4 hour period to assess beta-lactamase activity using the CENTA assay as described. A second study was performed using P2A (final concentration of 200 ng/mL) added to mixed chyme, mixed chyme supplemented with 0.1 mM ZnSO_4_, individual chyme samples, or pH-adjusted chyme 3 (original pH 5.58). Samples collected over a 6-hour period were assessed for beta-lactamase biological activity as described.

### 2.7. Fermentation and Purification of P2A Enzyme

An *E. coli* production cell line was generated using an optimized P2A expression plasmid, pET30a-P2A, containing the T7 promoter driving expression of the P2A coding region in the pET plasmid backbone (EMD Biosciences). The P2A expression plasmid was used to transform *E. coli* DE3 cells (New England Biolabs, Cat# C25271), and protein expression was induced by addition of 0.1 mM IPTG. Research cell bank stocks of the P2A cell line were produced and used to inoculate a 100 L batch fermenter. Cell pellets were collected and stored at −80 °C until processed.

A total of 2537 g of frozen cell paste was used to obtain 50 mg of P2A estimated at > 90% purity with 100% of expected biological activity. For P2A purification, cell paste (500 g per batch) was resuspended in 8L of Buffer A (20 mM Tris-Cl, pH 7.9, 0.1 mM ZnSO_4_) and homogenized with a Microfluidizer M110P. Lysate containing soluble P2A was harvested by centrifugation and clarified using a 0.8 µm filter. Lysate was loaded onto Q-sepharose Fast Flow resin XK-50 column equilibrated with one column volume (CV) of Buffer B (20 mM NaOAc, pH 5.1, 0.1 mM ZnSO_4_, and 1 M NaCl) followed by 2 CV Buffer A. Bound P2A was eluted with a linear gradient of 0% Buffer B to 100 % Buffer B in 8 CV. Peak fractions (50 mL/fraction) were collected, analyzed by SDS-PAGE and selected fractions were pooled. For the second chromatographic purification step, pooled fractions were loaded onto a diethylaminoethanol (DEAE) sepharose CL-6B column equilibrated with Buffer A, washed, and P2A eluted with Buffer B. Peak fractions were pooled and checked by SDS-PAGE. Biological activity was verified via the CENTA assay as described.

### 2.8. Formulation of P2A for Oral Delivery

Purified P2A protein was formulated for oral administration by incorporation into Eudragit^®^ (Evonik Industries AG, Darmstadt, Germany)-coated sucrose pellets designed to release active enzyme at pH 5.5 or greater as described [28], with the addition of a 7% hydroxypropylcellulose (HPC) isolation layer between the P2A and Eudragit^®^ coating to protect the P2A from the low pH (pH 2.8) Eudragit^®^ coating solution. Use of the HPC isolation layer resulted in the retention of full P2A biological activity. Gelatin capsules suitable for oral delivery were filled with the pellets for a total P2A content of 50 mg/capsule. Orally formulated P2A was renamed SYN-006.

For the dissolution analyses, the SYN-006 enteric-coated pellet formulation was held in a 0.1 N HCl solution for 2 h after which the pH was raised to 6.5. Samples collected at the indicated times over 6 h were assessed for kinetic enzyme dissolution by ultraviolet (UV) absorbance. Specifically, sample absorbance was read at 280 and 320 nm, and the reading was taken as A_280-320_ after subtracting the blank containing only dissolution buffer. Biological activity was evaluated using the CENTA assay as described [25].

## 3. Results

### 3.1. Beta-Lactamase Production, Purification, and Verification of Biological Activity

To identify carbapenemases with the potential to protect the gut microbiome from all classes of beta-lactam antibiotics, promising enzymes were selected based on published antibiotic degradation profiles, prioritizing enzymes that displayed the broadest activity spectrum. The class B metallo-beta lactamases, P2A [19] and NDM-1 [20], and the class A serine beta-lactamase, KPC-1 [21] were chosen for evaluation. A panel of *E coli* production strains was generated for each selected enzyme and screened for high-level protein production followed by verification of beta-lactamase biological activity. The highest expressing strains for each enzyme were subjected to 5L fermentation and chromatographic purification. Strong protein bands of the expected size, with little observable contamination with other proteins, were observed for all enzymes by SDS-PAGE (Figure S1A). Final yields of the enzymes, estimated to be >90% pure, were approximately 600 mg per liter. The specific activity of each enzyme was compared to that of ribaxamase in the CENTA assay (Figure S1B). The relative potencies of each enzyme for hydrolysis of CENTA were ribaxamase (100%) > NDM (71%) > KPC (33%) > P2A (12%). Notably, this potency ranking is based only on the hydrolysis kinetics of CENTA, a derivative of the first generation cephalosporin, cephalothin [25], and, therefore, enzyme activities are expected to differ with other antibiotic substrates. However, this assay demonstrates that all three carbapenemases could be expressed in *E. coli* and retained biological activity following chromatographic purification.

### 3.2. Antibiotic Degradation Profiles of Purified Carbapenemases

**Figure 1:**
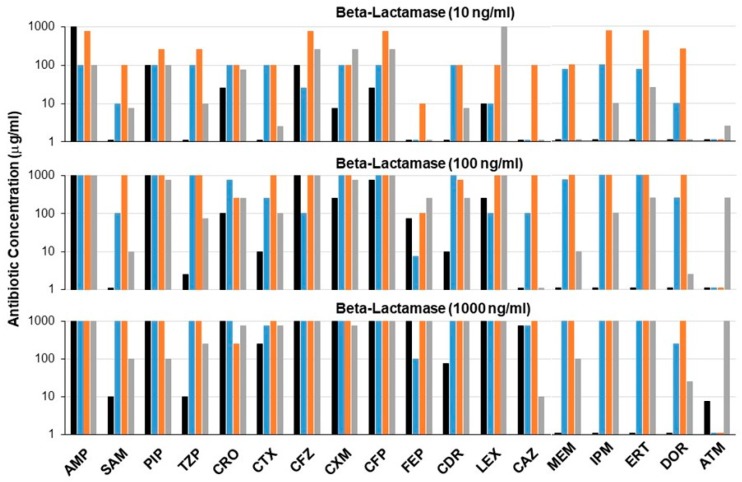
Antibiotic degradation profiles of beta-lactamase enzymes. Antibiotic degradation was assessed using an *E. coli* growth assay. Purified beta-lactamases (10, 100, or 1000 ng/mL) were added to wells of a microtiter plate containing high antibiotic concentrations (10, 100, or 1000 µg/mL) after which *E. coli* were added immediately. Bacterial growth, an indication of antibiotic inactivation, was measured the next day (OD625). The data represent the highest concentration of antibiotic that was inactivated by the beta-lactamase. Ribaxamase: black, P2A: blue, New Delhi metallo-beta-lactamase (NDM): orange, *Klebsiella pneumoniae* carbapenemase (KPC): gray. AMP: ampicillin, SAM: ampicillin/sulbactam, PIP: piperacillin, TZP: piperacillin/tazobactam, CRO: ceftriaxone, CTX: cefotaxime, CFZ: cefazolin, CXM: cefuroxime, CFP: cefoperazone, FEP: cefepime, CDR: cefdinir, LEX: cephalexin, CAZ: ceftazidime, MEM: meropenem, IPM: imipenem, ERT: ertapenem, DOR: doripenem, ATM: aztreonam.

To compare directly the antibiotic inactivation potential of each purified carbapenemase, a microtiter plate bacterial growth assay was used to mimic beta-lactamase activity in the gut in the presence of high antibiotic concentrations [5]. A panel of 18 antibiotics and antibiotic/beta-lactamase inhibitor combinations was evaluated. Low concentrations of purified P2A, NDM, KPC, and ribaxamase (10, 100, or 1000 ng/mL) were mixed with 1000-fold higher concentrations (10, 100, or 1000 µg/mL) of the indicated antibiotics (Figure 1). Beta-lactamase activity was determined as positive or negative based on bacterial growth, with growth indicating that the beta-lactamase inactivated the antibiotic. The bars on the graph represent the highest concentration of each antibiotic that was inactivated by the beta-lactamase (Figure 1). The carbapenemases P2A, NDM, and KPC, displayed a broader degradation profile than ribaxamase, notably activity against the carbapenems, meropenem, imipenem, ertapenem, and doripenem. NDM appeared to be the most potent beta-lactamase that efficiently degraded all tested cephalosporins and carbapenems. P2A displayed good activity against all carbapenems and most cephalosporins. Cefepime appeared most resistant to degradation by all beta-lactamases. Several cephalosporins including, cefazolin, cephalexin, and ceftazidime, were more efficiently degraded by NDM than by P2A; however, complete inactivation by P2A could be achieved by increasing enzyme concentration. KPC was the only beta-lactamase active against the monobactam, aztreonam. All three carbapenemases demonstrated some resistance to the beta-lactamase inhibitors, sulbactam and tazobactam, while ribaxamase showed activity in the presence of inhibitors only at the highest enzyme concentrations. Notably, NDM and P2A activity were unaffected by beta-lactamase inhibitors.

### 3.3. Stability Evaluation in Human Intestinal Fluid

**Figure 2:**
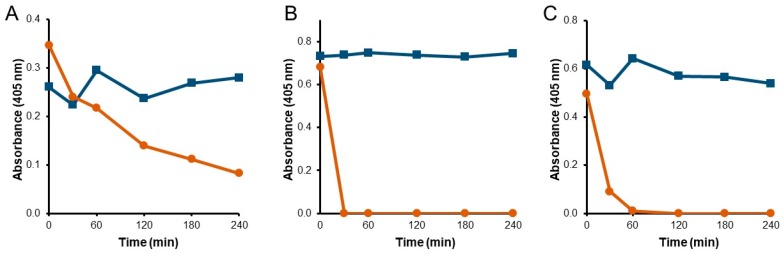
Functional stability of carbapenemases incubated with human intestinal chyme. P2A (**A**), NDM (**B**), or KPC (**C**), were incubated with buffer (blue squares) or mixed human chyme (orange circles). Samples were collected over 4 h and assessed for beta-lactamase activity.

Resistance to degradation by intestinal proteases is a key requirement for beta-lactamases intended for function in the GI tract. To assess the stability of P2A, NDM, and KPC, each carbapenemase was incubated with human intestinal chyme and evaluated for biological activity (Figure 2). While all three enzymes displayed stable activity when incubated in assay buffer, P2A was the only enzyme that retained biological activity for the four-hour collection period in chyme (Figure 2A). NDM immediately lost all activity by the first time point at 30 min (Figure 2B), and KPC lost activity completely within 60 min (Figure 2C).

**Figure 3:**
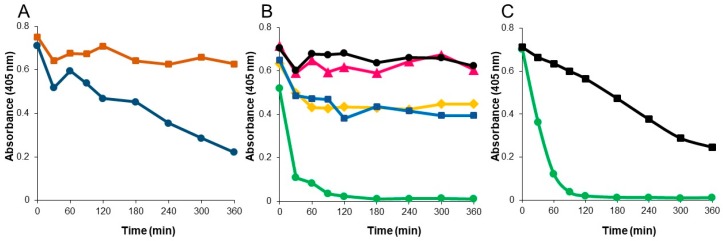
Functional stability of P2A incubated with human chyme under differing conditions. (**A**) P2A incubated with mixed human chyme supplemented with 0.1 mM ZnSO_4_ (orange squares) or without zinc (blue circles). (**B**) P2A incubated with human chyme samples collected from five different donors. Chyme 1: pink triangles, Chyme 2: black circles, Chyme 3: green circles, Chyme 4: yellow diamonds, Chyme 5: blue squares. (**C**). P2A incubated with Chyme 3, pH 5.6 (green circles) or pH-adjusted Chyme 3, pH 7.0 (black squares). Samples were collected over 6 h and assessed for beta-lactamase activity.

Although P2A retained function in mixed human chyme, its activity continued to decrease over the four hour incubation period (Figure 2A). P2A is a metallo-enzyme that requires zinc for substrate hydrolysis [23]. To determine if addition of zinc would stabilize P2A function, mixed chyme was supplemented with 0.1 mM ZnSO_4_ and P2A activity evaluated (Figure 3A). The presence of zinc resulted in a dramatic improvement in P2A functional stability in mixed chyme, with biological activity remaining stable over the six-hour incubation period, compared to a decline in activity in the same mixed chyme sample without zinc supplementation. Chyme was supplemented with 0.1 mM ZnSO_4_ for all subsequent analyses.

Evaluation of P2A in the individual chyme samples demonstrated that P2A function remained stable in four of the five chyme samples over the six-hour incubation period (Figure 3B). However, activity rapidly declined to undetectable by 2 h in chyme sample 3. To determine why P2A was unstable in chyme sample 3, the chyme samples were characterized by liquid content (55% to 78%), protease activity (5.57 to 8.96 mU/mL), and pH (5.58 to 6.56) (Table S1). Notably, chyme sample 2 displayed the highest protease concentration (8.96 mU/mL) while chyme sample 3 had the lowest pH (5.58) of the five samples. To test if P2A functional stability was influenced by pH, the pH of chyme sample 3 was adjusted to 7.0, and P2A biological activity reassessed (Figure 3C). While P2A functional stability was dramatically improved in pH-adjusted chyme sample 3, enzymatic activity declined steadily to 50% over 6 h, suggesting that another factor, in addition to pH, influenced P2A stability in chyme sample 3. As P2A was the only carbapenemase that retained antibiotic degradation activity in human chyme, P2A was chosen as the candidate with the best potential for clinical efficacy.

### 3.4. Optimal pH Range for Peak P2A Biological Activity

**Figure 4:**
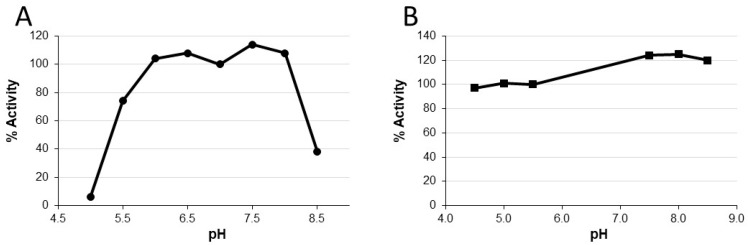
P2A biological activity under differing pH conditions. (**A**) P2A was incubated in assay buffer at the indicated pH for 15 min, prior to activity assessment. (**B**) P2A was incubated for 24 h at 4 °C in buffers of the indicated pH, after which the pH was raised to 7.5 and activity assessed.

P2A biological activity declined rapidly in chyme sample 3 at pH 5.6 and functional stability was improved when the pH of the sample was raised to 7.0, indicating that P2A activity is pH-dependent. To determine the pH range for optimal P2A function, the CENTA activity assay [25] was performed in pH-adjusted buffers, ranging from pH 5.0 to pH 8.5 in 0.5 pH unit increments. The P2A CENTA assay is normally performed in pH 7.0 phosphate buffer with supplementation with 0.1 mM ZnSO_4_. Compared to the P2A standard curve, the relative P2A activity in each pH-adjusted buffer was calculated (Figure 4A). Peak activity occurred between pH 6.0 to 8.0, with no activity detected at pH 5.0, and approximately 40–50% peak activity at pH 8.5 and pH 5.5, respectively.

To determine if P2A could regain biological activity after prolonged incubation at low pH, a similar study was performed where P2A was incubated in buffers with pH of 4.5, 5.0, 5.5, 7.5, 8.0, and 8.5 for 24 h at 4 ℃, after which the P2A samples were pH adjusted by dilution in pH 7.5 assay buffer prior to CENTA analysis. The relative P2A activities were calculated by comparison to a P2A standard curve (Figure 4B). P2A relative activities ranged from 97% to 125% indicating that P2A could regain full function following prolonged incubation at pHs as low as 4.5. These data demonstrate that peak P2A activity is between pH 6.0 to 8.0, P2A activity is compromised at pHs at or below 5.0 and at or above 8.5, and that function can be restored after extended low pH exposure by increasing pH to within the peak activity range.

### 3.5. P2A Formulation into Enteric-Coated Enzyme Pellets and In Vitro Dissolution Analyses

P2A production was scaled up in a 100 liter fermenter and purified using a, two step chromatographic method. CENTA analysis verified that the >90% pure P2A protein retained full biological activity. A total of 50 mg of P2A protein was obtained from approximately 2.5 kg of bacterial cell paste. Purified P2A protein was formulated for oral delivery by incorporation into Eudragit^®^-coated sucrose pellets designed to release active enzyme at pH 5.5 or greater [28]. Because P2A is sensitive to low pH, and the Eudragit^®^ coating solution is pH 2.8, inclusion of a 7% HPC isolation layer was required after P2A layering onto the sucrose pellets prior to coating with Eudragit^®^ to protect the P2A from the Eudragit^®^ coating solution. Use of the HPC isolation layer resulted in retention of full P2A biological activity. Formulated P2A was renamed SYN-006.

**Figure 5:**
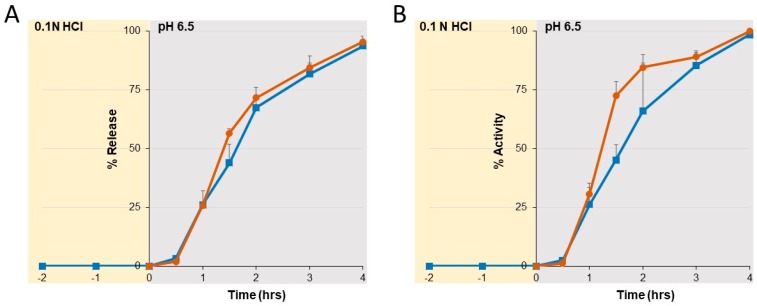
Dissolution of enteric-coated pellets containing P2A (SYN-006). SYN-006 pellets, composed of enteric-coated P2A, were held at low pH (0.1 N HCl) for two h after which the pH was raised to 6.5 (blue squares) or were exposed to pH 6.5 buffer alone (orange circles). (**A**) Kinetic enzyme dissolution (% release) of P2A enzyme from SYN-006 pellets assessed by ultraviolet (UV) absorbance. (**B**) Activity (%) of P2A enzyme released from SYN-006 pellets.

To evaluate the dissolution characteristics of SYN-006, studies were conducted with incubation at low pH followed by increasing the pH (Figure 5). Enzyme release (Figure 5A) was compared to enzyme activity (Figure 5B). No enzyme was released during low pH incubation, indicating that the SYN-006 pellets remained intact. Dissolution initiated within a half an hour of exposure to the pH 6.5 buffer with enzyme released completely by 4 h. Percent release compared well with percent activity, indicating that P2A retained full biological activity after dissolution. In addition, curves were similar for samples preincubated in 0.1 N HCl to those exposed only to pH 6.5 buffer, verifying that the SYN-006 enteric-coated formulation protected P2A from inactivation at low pH. These dissolution data confirm that the SYN-006 enteric coating remained intact at low, stomach pH, and that fully functional P2A enzyme was released at pH 6.5, similar to pH levels found in the small intestine.

### 3.6. P2A Enzyme Kinetics

In addition to evaluating the antibiotic degradation potential of P2A using the bacterial growth assay (Figure 1), kinetic parameters of antibiotic hydrolysis were determined for P2A and compared to those of ribaxamase (Table 1). The beta-lactam antibiotics tested included penicillins, cephalosporins, and carbapenems. The k_cat_/K_M_ values were well over 1000 for the vast majority of antibiotics for both ribaxamase and P2A. As expected, ribaxamase had no activity with the carbapenems, imipenem and meropenem, while P2A displayed k_cat_/K_M_ values of well over 1000 for both carbapenems. Relative potency of P2A and ribaxamase with each antibiotic as determined from the bacterial growth assay (Figure 1) were consistent with the kinetic analyses based on the k_cat_/K_M_ calculations. For example, ribaxamase displayed more efficient degradation of ampicillin than P2A in the bacterial growth assay, and the k_cat_/K_M_ value for ribaxamase was approximately 10-fold higher than that for P2A. Likewise, cefotaxime was more efficiently degraded by P2A than ribaxamase in the bacterial growth assay, and the P2A k_cat_/K_M_ was approximately 4-fold higher than ribaxamase. Notably, antibiotics that displayed low k_cat_/K_M_ values, such as P2A with cefazolin (154 k_cat_/K_M_) and ribaxamase with cefuroxime (126 k_cat_/K_M_), were efficiently hydrolyzed at high enzyme concentrations in the bacterial growth assay (Figure 1).

## 4. Discussion

Antibiotics damage the gut microbiome and provide selective pressure for the emergence of antibiotic resistance. Broad spectrum beta-lactam antibiotics, including penicillins, cephalosporins, and carbapenems, are especially damaging to the gut microbiome [6,29,30] and constitute a major risk factor of *C. difficile* infection [15]. In animal and human studies, oral administration of a beta-lactamase enzyme, ribaxamase, was demonstrated to degrade IV ceftriaxone in the GI tract, protect the gut microbiome, attenuate emergence of antibiotic resistance, and significantly decrease *C. difficile* infection [5,6,7,8,9,10].

Ribaxamase degrades penicillin and most cephalosporin antibiotics [5], but it does not inactivate carbapenems. To expand this microbiome protection strategy to include all classes of beta-lactams, three distinct carbapenemases were screened with a focus on manufacturability, antibiotic degradation spectrum, and stability in human intestinal fluid. All three carbapenemases were expressed at high levels in *E. coli* production cell lines and retained biological activity following purification. Additionally, the carbapenemases displayed efficient degradation of all classes of beta-lactam antibiotics, including carbapenems and antibiotic/beta-lactamase inhibitor combinations. Surprisingly, functional stability in human chyme, a key requirement for efficacy of an orally-delivered enzyme, became the defining characteristic for the three candidates. P2A was the only carbapenemase that displayed sustained biological activity in human chyme, and was, therefore, chosen as a potential clinical candidate. Notably, ribaxamase also displayed prolonged function when incubated with human chyme [5], suggesting that this in vitro chyme stability assay may be predictive of clinical potential.

An explanation for the capacity of P2A and ribaxamase to avoid digestion when incubated with human chyme, compared to the rapid inactivation of NDM and KPC, may lie in the bacterial origin of these enzymes. P2A is derived from *Bacillus cereus* [19], and ribaxamase, an engineered derivative of the PenP enzyme, was isolated from *Bacillus licheniformis* [5]. *Bacillus* are Gram-positive bacteria. In contrast, NDM and KPC originate from Gram-negative species, specifically *Escherichia coli* [20] and *Klebesiella pneumoniae* [21]. The cell wall structures of Gram–positive and Gram-negative organisms differ markedly, as Gram-negative bacteria have, in addition to an inner membrane adjacent to the cytoplasm, another membrane outside the cell wall that is lacking in Gram-positive organisms. Gram-negatives secrete beta-lactamases and many other proteins, into the periplasmic space, the area between the two membranes, where beta-lactamases function to degrade beta-lactams and confer antibiotic resistance [31]. The outer membrane serves as a protective barrier [31], sheltering periplasmic beta-lactamases from the outside environment. However, Gram-negatives have evolved a means to disseminate periplasmic proteins extracellularly through encapsulation within outer membrane vesicles (OMV). For example, NDM secreted within OMV was demonstrated to retain biological activity and to confer carbapenem resistance to proximal susceptible bacteria [32]. In contrast, beta-lactamases secreted by Gram-positive organisms are exposed directly to the extracellular environment, and thus appear to have evolved enhanced conformational stability to retain function under these harsh conditions, such as the lumen of the mammalian GI tract. Regardless of why P2A and ribaxamase display extreme functional stability, resistance to degradation by intestinal proteases is a key attribute for beta-lactamases intended for GI-targeted therapy.

Another important characteristic of a beta-lactamase clinical candidate is the ability to inactivate a broad range of beta-lactam antibiotics in the GI tract. In this study, antibiotic degradation was evaluated with an *E. coli* growth microtiter plate assay that employed bacterial growth as the indicator for antibiotic degradation [5]. The three carbapenemases efficiently degraded all classes of beta-lactam antibiotics. In general, Michaelis–Menten enzyme kinetics were consistent with bacterial growth assay results. The low k_cat_/K_M_ ratio calculated for P2A with cefazolin (154 k_cat_/K_M_) suggested inefficient antibiotic hydrolysis and was supported by the observation that cefazolin was more efficiently degraded by ribaxamase (5189 k_cat_/K_M_) than P2A in the bacterial growth assay. However, complete inactivation of cefazolin could be achieved by increasing P2A concentration. Likewise, ribaxamase displayed unfavorable kinetics for cefuroxime hydrolysis (126 k_cat_/K_M_) compared to P2A (8185 k_cat_/K_M_). Bacterial growth was achieved with 10 ng/ml of P2A while ribaxamase required 1000 ng/ml for bacterial growth. These data suggest that complete inactivation of beta-lactam antibiotics is achievable with high beta-lactamase concentrations, including those antibiotics that display unfavorable k_cat_/K_M_ degradation kinetics. The bacterial growth assay, designed to model in vivo microbiota protection via antibiotic inactivation [5], may more closely model antibiotic degradation requirements in the GI tract, where both antibiotic and enzyme concentrations are expected to be high. Indeed, ribaxamase concentrations >1,000,000 ng/mL were measured in the upper GI tract of some individuals [9]. These data suggest that P2A has the potential to degrade a broad spectrum of beta-lactam antibiotics at achievable enzyme concentrations in the GI tract.

Characterization of P2A revealed improvement of functional stability in the presence of zinc and that P2A activity was pH dependent. Peak activity occurred between pH 6-8, and function was lost at pH 5.0 and 8.5. Notably, P2A activity could be restored by adjusting the pH to within its functional range in the presence of zinc. These two seemingly disparate observations can be explained by P2A enzyme structure. P2A is a class B1 metallo-beta-lactamase [23] that requires two zinc ions in its binuclear active site for antibiotic binding and beta-lactam ring hydrolysis [33]. P2A shares 93% amino acid homology to BCII, a well-studied class B1 metallo-enzyme also isolated from *B. cereus* [34]. Evaluation of BCII function at pH ≤ 5.5 demonstrated protonation of the metal-binding ligands resulting in zinc ion release and loss of function [35], suggesting that P2A activity loss at low pH was caused by a similar mechanism. Therefore, similar to BCII, subsequent increase of pH in the presence of zinc most likely restored P2A metal ion binding capacity resulting in recovery of full biological activity. Notably, metal deprivation not only hampers metallo-beta-lactamase activity but also affects protein stability [32], suggesting that without zinc, a conformational change occurs, most likely loosening of tertiary structure, rendering the enzyme vulnerable to degradation. These data may help to explain P2A activity loss upon incubation in human chyme sample 3 (pH 5.58). With no proteases present, such as in the low pH buffer studies, P2A polypetide integrity remained intact, allowing the enzyme to refold with zinc and regain full activity at optimal pH. In contrast, in the presence of proteases in human chyme at low pH, P2A became structurally accessible to degradation resulting in permanent activity loss. Indeed, increasing the pH of chyme 3 dramatically improved P2A functional stability; however, P2A activity continued to decline over the six hour incubation suggesting that an additional agent, perhaps a specific protease detrimental to P2A function, was present. As P2A activity remained stable in the mixed chyme samples containing 20% chyme 3, the negative effects of this hypothesized protease were overcome by dilution.

The requirement for zinc and sensitivity to low pH is not expected to limit P2A clinical utility. For enzyme production, P2A biological activity was preserved by including zinc in all media and buffers, and, for SYN-006 formulation, an isolation layer was positioned between P2A cores and the pH 2.8 enteric coating solution to protect the enzyme from low pH during enzyme pellet production. Therefore, to ensure sufficient zinc availability in the GI tract, SYN-006 could be formulated with added zinc and/or patients could be advised to take a zinc supplement with each SYN-006 dose. Similarly, proton pump inhibitors (PPIs) which raise the pH of intestinal fluid could be prescribed with SYN-006 during the course of antibiotic treatment. The use of PPIs is not expected to impact P2A function negatively as PPIs had no effect on ribaxamase antibiotic degradation efficacy in a Phase 2a clinical study [9].

SYN-006 is primed for evaluation in an established porcine model of carbapenem-mediated gut dysbiosis [29] to determine if SYN-006 can protect the gut microbiome from carbapenem collateral damage and reduce the emergence of antibiotic resistance. SYN-006 has the potential to expand microbiome protection via antibiotic inactivation in the GI tract to all classes of beta-lactam antibiotics, including carbapenems.

## Figures and Tables

**Table 1:** Antibiotic degradation kinetics of ribaxamase and P2A. Steady-state K_M_ and k_cat_ values for the indicated beta-lactam antibiotics were determined by measuring substrate hydrolysis under initial rate conditions with Hanes linearization of the Michalis-Menten equation. Relative antibiotic degradation efficiency based on the *E. coli* growth assay is displayed in the last column for comparison.

Antibiotic	SYN-004 (Ribaxamase)			P2A			*E. coli* Assay
	K_M_	K_cat_	K_cat_/K_M_	K_M_	K_cat_	K_cat_/K_M_	
	mM	s^−1^	M^−1^ s^−1^ (× 10^−3^)	mM	s^−1^	M^−1^ s^−1^ (× 10^−3^)	
Ampicillin	161	2160	13417	942	1114	1183	ribaxamase > P2A
Pipercillin	53	816	15396	372	1049	2820	ribaxamase = P2A
Ceftriaxone	38	83	2184	68	95	1397	P2A > ribaxamase
Cefotaxime	21	36	1714	66	479	7258	P2A > ribaxamase
Cefazoline	37	192	5189	39	6	154	Ribaxamase > P2A
Cefoperazone	2	17	8500	82	338	4122	P2A > ribaxamase
Cefuroxime	277	35	126	27	221	8185	P2A > ribaxamase
Imipenem	Not active	Not active		337	1480	4392	P2A
Meropenem	Not active	Not active		410	480	1171	P2A

## Supplementary Materials

The following are available online at http://www.mdpi.com/2076-2607/7/1/22/s1, Figure S1: Purification and characterization of the carbapenemases, P2A, NDM, and KPC. Table S1: Human chyme analyses. Chyme was collected from five donors with ileostomies and was characterized based on pH, liquid content, and protease activity.
