# Supplementary material for: Identification, Characterization, and Formulation of a Novel Carbapenemase Intended to Prevent Antibiotic-Mediated Gut Dysbiosis
Source: Microorganisms. 2019 Jan 16;7(1):22. doi: 10.3390/microorganisms7010022 (PMC6352093; doi:10.3390/microorganisms7010022)
Supplement: Supplementary file 1 [file microorganisms-07-00022-s001.pdf]

Identification, Characterization, and Formulation of a Novel Carbapenemase Intended to Prevent Antibiotic-Mediated Gut Dysbiosis

Sheila Connelly *, Todd Parsley, Hui Ge, and Michael Kaleko

Table S1. Human chyme analyses. Chyme was collected from five donors with ileostomies and was characterized based on pH, liquid content, and protease activity. Mixed Chyme contained equal volumes of each of the five chyme samples.

Specimen	pH	% Liquid	Protease Activity (mU/mL)
Chyme 1	6.42	55	5.57
Chyme 2	5.98	57	8.96
Chyme 3	5.58	57	6.63
Chyme 4	6.26	66	6.21
Chyme 5	6.56	78	6.56
Mixed Chyme	6.20	68	6.13

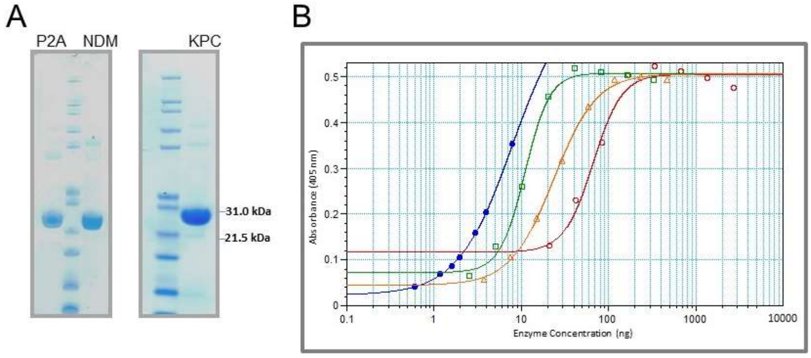

Figure S1. Purification and characterization of the carbapenemases, P2A, NDM, and KPC. (**A**) SDA/PAGE aanlysis of purified P2A, NDM, and KPC proteins. (**B**) Biological activity assessment using CENTA reagent. Blue closed circle: ribaxamase, green open square: NDM, orange open triangle: KPC, red open circle: P2a. Relative activity with CENTA substrate: ribaxamase (1.00) > NDM (0.71) > KPC (0.33) > P2A (0.12).
